# Effects of Estradiol and Progesterone-Induced Intracellular Calcium Fluxes on *Toxoplasma gondii* Gliding, Microneme Secretion, and Egress

**DOI:** 10.3389/fmicb.2018.01266

**Published:** 2018-06-12

**Authors:** Xiao Zhang, Heng Zhang, Yong Fu, Jing Liu, Qun Liu

**Affiliations:** ^1^National Animal Protozoa Laboratory, College of Veterinary Medicine, China Agricultural University, Beijing, China; ^2^Key Laboratory of Animal Epidemiology of the Ministry of Agriculture, College of Veterinary Medicine, China Agricultural University, Beijing, China

**Keywords:** estradiol, progesterone, *Toxoplasma*, calcium intracellular release, gliding, microneme secretion, egress

## Abstract

Research has shown that estrogen is present and plays a critical role in vertebrate reproduction and metabolism, but the influence of steroids on *Toxoplasma gondii* has received less attention. Our data showed that estradiol and progesterone induced parasitic cytosolic Ca^2+^ fluxes. This process required estrogen to enter the cytoplasm of *T. gondii*, and cGMP-dependent protein kinase G (PKG) and phosphoinositide-phospholipase C (PI-PLC) emerged as important factors controlling parasitic intracellular (IC) Ca^2+^ signals. Cytosolic Ca^2+^, which is regulated by estradiol, was mostly mobilized from acidic organelles. Moreover, cytosolic Ca^2+^ slightly increased MIC2 protein secretion and promoted the gliding motility and egress of parasites, thus enhancing the pathogenicity of *T. gondii*, as shown in our previous research. We subsequently determined that the main source of Ca^2+^ regulated by progesterone was a neutral store. In contrast to the findings of estradiol, progesterone reduced MIC2 protein secretion and inhibited the gliding motility of parasites, which may decrease their pathogenicity. Additionally, unlike in mammals, estradiol and progesterone had no effect on nitric oxide (NO) or reactive oxygen species (ROS) production in *T. gondii*.

## Introduction

*Toxoplasma gondii* is found worldwide, and this pathogen can infect a wide range of hosts, such as humans, livestock, pets, and wildlife. All karyocytes are considered to be definitive cells of *T. gondii* (Montoya and Liesenfeld, 2004). As an opportunistic parasite, *T. gondii* has been held responsible for high morbidity in some special cases, such as the high transmission frequency of *T. gondii* observed during pregnancy (Roberts et al., 2001). Concentrations of estradiol and progesterone are several-fold higher in pregnant females than in non-pregnant females, and the infection rate of pathogens is also significantly increased. However, the fact that pathogens can utilize sex steroid hormones for their survival and reproductive success is often overlooked (Vom Steeg and Klein, 2016).

In mammals, hormone effects are divided into short-term actions (seconds to minutes) and long-term actions (hours to days), which depend on different receptors. There are currently no available reports concerning *T. gondii* estrogen receptors. Although our research group has attempted to explore nuclear receptors (mediating long-term actions) of *T. gondii* for quite some time, the results have been inconclusive. Therefore, the study of estrogen-mediated rapid effects is a different approach for obtaining an in-depth understanding of this topic. Short-term actions, such as membrane-and/or cytosol-initiated mechanisms, involve a rapid response through the activation of signal transduction pathways mediated by membrane estrogen receptors (mERs) or G-protein-coupled receptors (GPCRs) and ion channels, modulating kinase activation and ionic fluxes, such as calcium fluxes (Vasudevan and Pfaff, 2008; Mcewen et al., 2012). Because calcium and hormone membrane-initiated mechanisms have physiological implications relevant to other organisms as non-transcriptional mechanisms (Vega-Vela et al., 2017), the possible interaction between estrogen and calcium fluxes in *T. gondii* is a topic deserving of discussion.

The life cycle of *T. gondii* is accompanied by fluxes of cytosolic Ca^2+^. These fluxes are necessary for parasite motility and egress from host cells. During the *T. gondii* life cycle, the parasite invades host cells to create a parasitophorous vacuole (PV), where it divides and matures. Parasite development involves several steps: (i) gliding motility, (ii) conoid extrusion, (iii) secretion of specific proteins, (iv) attachment to the host cell, (v) active invasion, and (vi) egress. Previous studies have shown that intracellular (IC) Ca^2+^ fluxes are important for the initiation of gliding motility, microneme secretion, conoid extrusion, active parasite invasion, and egress (Pu and Zhang, 2012). Our previous work showed that estradiol could promote the invasion and proliferation of *T. gondii* and thus significantly contribute to the pathogenicity of *T. gondii* in mice (Zhang et al., 2017). Therefore, Ca^2+^ induced by estradiol in these processes may be one factor in estrogen-promoted high pathogenicity.

Genetically encoded Ca^2+^ indicators (GCaMPs) were used to develop a parasite strain for the observation of estrogen-induced Ca^2+^ signals in *T. gondii*. Our results showed that estradiol and progesterone induce the release of Ca^2+^ of *T. gondii* from different stores. Additionally, estrogen-induced Ca^2+^ fluxes relate to parasite gliding motility, microneme secretion, and egress.

## Materials and Methods

### Parasites and Cell Culture

HFFs (human foreskin fibroblasts) and Vero cells (African green monkey kidney cells) were obtained from the Cell Bank of the Chinese Academy of Sciences (Shanghai, China). *T. gondii* RH strain tachyzoites (provided by Xingquan Zhu, Chinese Academy of Agricultural Sciences) and the RH-GCaMP6f strain (provided by Silvia N. J. Moreno, University of Georgia) were maintained *in vitro* on Vero or HFF cells in DMEM (M&C, China) containing 25 mM glucose and 4 mM glutamine supplemented with 8% fetal bovine serum (FBS, Gibco, United States) and were incubated at 37°C with 5% CO_2_ in a humidified incubator. The medium was changed 12 h after inoculation. The RH-GCaMP6f strain was constructed and provided by Silvia N. J. Moreno, and the detailed plasmid information was reported previously (Borgespereira et al., 2015). Briefly, plasmids for the expression of GCaMP6 in *T. gondii* were kindly provided by David Sibley at Washington University. The coding DNA sequence for GCaMP6f (Addgene) was amplified via PCR and cloned into a *T. gondii* vector for expression under the tubulin promoter.

### Measurement of Intracellular Oxidative Activity

Intracellular reactive oxygen species (ROS) levels in *T. gondii* were measured using the probe 2′, 7′-dichlorofluorescein diacetate (DCF-DA, Sigma, United States). Tachyzoites were pretreated with estrogen for different times and then harvested, after which they were incubated with 10 μM DCF-DA for 1 h at 37°C, washed twice with phosphate-buffered saline (PBS), and quantified utilizing flow cytometry. Estrogen-pretreated parasites using same method were used to measure NO activity in a chemiluminescence assay according to the manufacturer’s instructions (Jiancheng, China).

### Microneme Secretion Assay

Fresh tachyzoites were harvested, washed twice with PBS, and resuspended in extracellular (EC) buffer (1 mM MgCl_2_, 142 mM NaCl, 25 mM HEPES, 5 mM KCl, 5.6 mM D-glucose, 1.8 mM CaCl_2_, pH 7.4). Estradiol (Sigma, E8875, United States), and progesterone (Sigma, P0130, United States) were added to the resuspension solution, and parasites were allowed to secrete for 15 min at 37°C. After centrifugation at 2500 rpm, the supernatant and pellet were collected for western blot analysis.

### Cytosolic Ca^2+^ Measurements

For time-resolved microscopy, purified RH-GCaMP6f parasites in IC buffer (142 mM KCl, 5 mM NaCl, 2 mM EGTA, 5 mM MgCl_2_, 25 mM HEPES-KOH pH 7.2, 1 mg/ml BSA) were added to glass-bottom culture dishes and then heated to 37°C. Compounds were then added, and fluorescence images (3 min duration) were collected on an Olympus fluorescence microscope. The images were exported from Zen, and fluorescence intensities in the GFP channel were quantified in Image-Pro for each time slice.

### Egress Assay

Egress assays were performed as previously described (Morlonguyot et al., 2014). Fresh tachyzoites were harvested and added to HFF cells in a 12-well plate for 24 h culture. Then, the tachyzoites were washed with PBS, and dimethyl sulfoxide (DMSO, Thermo Fisher Scientific, United States), estradiol or progesterone diluted in DMEM was added followed by incubation for 30 min. An equivalent amount of DMSO was used as a solvent control. Parasites were labeled with anti-GAP45 antibodies (prepared in our laboratory), and the proportion of egressed versus non-egressed vacuoles was calculated by counting 100 vacuoles in triplicate from three independent biological replicates. Live images were obtained via microscopy.

### Gliding Assay

Freshly egressed RH-GCaMP6f tachyzoites were collected, purified, and lightly adhered to coverslips on ice for 4 min. Non-adhered parasites were washed away. The coverslips were then incubated with different reagents at 37°C for 15 min. Gliding conditions were quantified by measuring trails stained with a SAG1 antibody (prepared in our laboratory), and immunofluorescence images were acquired as previously described (Wetzel et al., 2004). For all treatments, at least 50 parasite trails were measured on each experiment.

### Statistical Analysis

Statistical significance between groups was evaluated by two-tailed unpaired Student’s *t*-tests using GraphPad Prism 5 (San Diego, CA, United States). Statistical data are presented as the mean value ± standard error of the mean (SEM). *P* < 0.05 and *P* < 0.01 were considered statistically significant and very significant, respectively.

## Results

### GCaMP6f-Expressing Tachyzoites Detect Estrogen-Induced Ca^2+^ Fluxes

**FIGURE 1:**
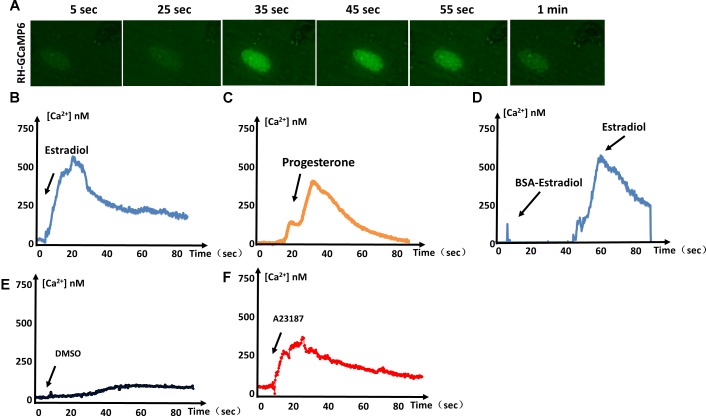
Estrogen-induced Ca^2+^ fluxes in *T. gondii.* **(A)** Video microscopy of intracellular parasites expressing GCaMP6f, following the addition of estradiol at 0 s. **(B)** Intracellular Ca^2+^ concentrations monitored over time in GCaMP6f-RH parasites (extracellular tachyzoites) stimulated with 10^-8^ M estradiol. **(C)** Intracellular Ca^2+^ fluxes of GCaMP6f-RH parasites were monitored after stimulation with 10^-5^ M progesterone. **(D)** GCaMP6f-RH parasites stimulated with estradiol after treatment with BSA-coupled estradiol. **(E)** DMSO was used as a negative control because all compounds dissolved in DMSO. **(F)** A23187 (calcium ionophore, 2 μM, Sigma, United States), a mobile ion-carrier, was used as a positive control for the monitoring of intracellular Ca^2+^ concentrations. Each experiment was performed in triplicate with three independent biological replicates.

GCaMP is generated through fusion of green fluorescent protein (GFP), calmodulin, and M13 (a peptide sequence from myosin light chain kinase). When calcium is present, calmodulin undergoes a conformational change and binds M13 to generate green fluorescence. To determine whether estradiol and progesterone induce an increase in Ca^2+^ release from parasitic IC stores, we used the genetically encoded Ca^2+^ biosensor GCaMP6s to monitor IC Ca^2+^ fluxes (**Figure 1A**). We observed green fluorescence representing the flux of increased Ca^2+^ in response to estradiol (**Figure 1B**) and progesterone (**Figure 1C**). Our previous studies have shown that BSA-coupled estradiol cannot enter the cytoplasm of *T. gondii* (Zhang et al., 2017). In **Figure 1D**, it can be seen that BSA-coupled estradiol failed to stimulate Ca^2+^ fluxes, which may indicate that there is no membrane receptor on the surface of *T. gondii* for estradiol binding to induce Ca^2+^ release. Of course, we cannot rule out a possible surface receptor for estradiol because BSA conjugation may have impaired estradiol binding to a potential receptor at its binding site.

### Ca^2+^ Stores Mobilized by Estrogen

**FIGURE 2:**
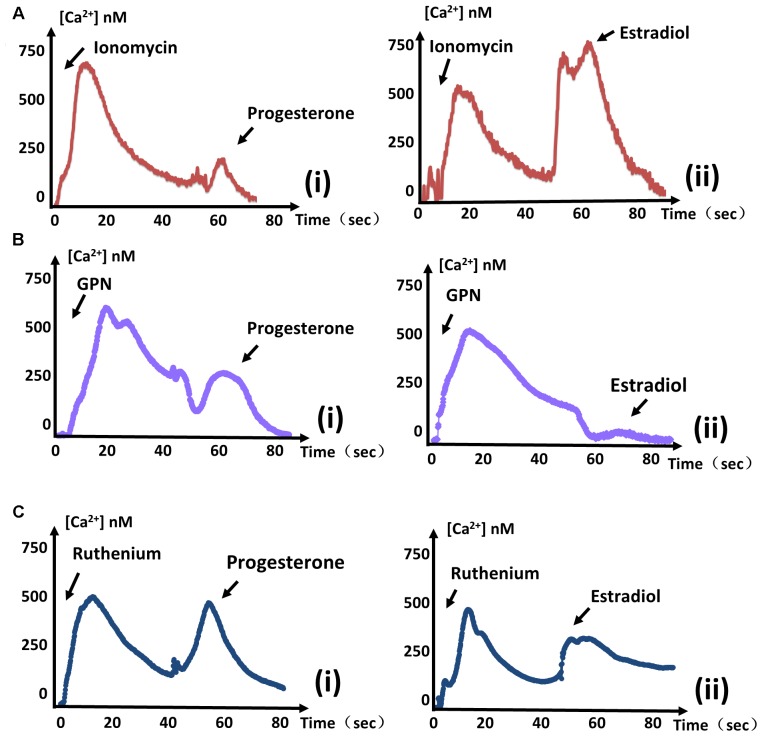
Estradiol-induced Ca^2+^ and progesterone-induced Ca^2+^ originate from different stores. **(A)** The same measurements were performed as in **Figure 1**. Ionomycin was added at 0 s, and progesterone **(i)** or estradiol **(ii)** was added at 60 s to monitor intracellular Ca^2+^ concentrations. **(B)** The intracellular calcium concentrations of parasites were measured after treatment with GPN at 0 s. Progesterone **(i)** or estradiol **(ii)** was added at 60 s, as indicated. **(C)** GCaMP6f-RH parasites were treated with ruthenium red at 0 s, and stimulated with progesterone **(i)** or estradiol **(ii)** at 40 s to observe Ca^2+^ fluxes. Each experiment was performed in triplicate with three independent biological replicates.

In general, *T. gondii* exhibits several regular Ca^2+^ stores, such as the endoplasmic reticulum (ER), mitochondria, and some acidocalcisomes. Ionomycin has been reported to specifically mobilize neutral Ca^2+^ stores of *T. gondii* (Moreno and Zhong, 1996). A peak in cytosolic Ca^2+^ levels was observed following ionomycin (100 μM, Sigma, United States) treatment, indicating that Ca^2+^ had been mobilized from neutral stores. Subsequent treatment with progesterone no longer produced an obvious Ca^2+^ peak, meaning that most of the progesterone-mobilized Ca^2+^ had already been depleted by ionomycin. Therefore, progesterone-induced Ca^2+^ mostly originates from neutral stores. In contrast, treating parasites with ionomycin followed by estradiol still produced an obvious Ca^2+^ spike (**Figure 2Aii**). These results mean that estradiol-induced Ca^2+^ does not originate from neutral stores, in contrast to progesterone.

Acidic Ca^2+^ stores are widely recognized as playing important roles in Ca^2+^ signaling. The addition of glycyl-L-phenylalanine-naphthylamide (GPN, 100 μM, Santa Cruz, United States) has a swelling effect on the release of Ca^2+^, and cause Ca^2+^ to leak from lysosomal-like compartments (Miranda et al., 2010). We treated parasites with GPN before estradiol and found that estradiol largely failed to induce a Ca^2+^ flux again (**Figure 2Bii**). In contrast, progesterone still induced a large quantity of Ca^2+^ release, regardless of the treatment (**Figure 2Bi**). Therefore, estradiol-induced Ca^2+^ is mostly derived from the acidic store of *T. gondii*, in contrast to progesterone.

Ca^2+^ influx into the mitochondrial matrix is mainly mediated by a Ruthenium compound-sensitive Ca^2+^ influx uniporter (O’Rourke, 2007), which has been used to reveal mitochondrial Ca^2+^ dynamics in *Plasmodium falciparum* (Rotmann et al., 2010). Ruthenium Red (RuRed, 10 μM, Meilunbio, China) failed to prevent the increase in Ca^2+^ concentration, regardless of whether it was induced by progesterone or estradiol (**Figure 2C**), indicating that increases of Ca^2+^ induced by the two estrogens did not originate from the mitochondrial matrix.

### Intracellular Ca^2+^ Fluxes Depend on PKG and PI-PLC

**FIGURE 3:**
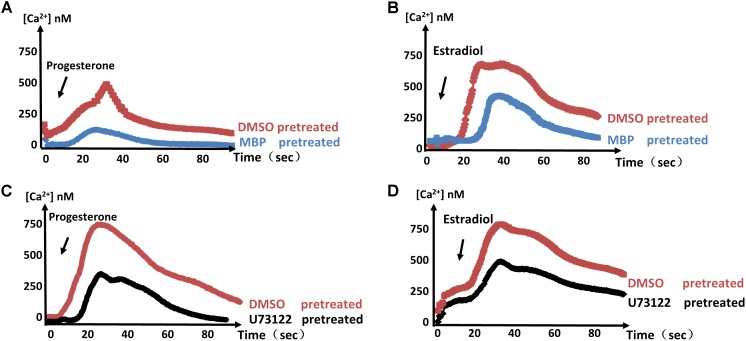
PKG and PI-PLC are important factors in the control of parasite Ca^2+^ signals. **(A,B)** Calcium concentrations in intracellular parasites were measured after stimulation with progesterone **(A)** or estradiol **(B)** in the presence of 100 μM MBP146-8. **(C,D)** 100 μM U73122 was used to verify that PI-PLC is necessary for the progesterone- **(C)** or estradiol-induced **(D)** Ca^2+^ spike. Each experiment was performed in triplicate with three independent biological replicates.

Previous studies have shown that cGMP-dependent PKG is essential to Ca^2+^ signals in malaria parasites (Brochet et al., 2014). MBP146-78 (MCE, United States) is a specific inhibitor of PKG and displays dose-dependent inhibition of PKG in *T. gondii* tachyzoites (Bakela et al., 2002). To determine whether estrogen influences Ca^2+^ in parasites through PKG, we measured the responses of estradiol and progesterone after MBP146-78 (200 nM) treatment, and a reduced Ca^2+^ spike was observed compared with the control group (**Figures 3A,B**). These results showed that the estradiol and progesterone-induced Ca^2+^ spikes may be related to PKG.

U-73122 (MCE, United States) is a phosphoinositide-phospholipase C (PI-PLC)-specific inhibitor. Using the same method, we found that PI-PLC is required for the estrogen-induced Ca^2+^ spikes (**Figures 3C,D**). These results indicate that PKG and PI-PLC are important factors in estrogen-induced calcium production. Of course, we cannot rule out the possible existence of other factors or signaling pathways that mediate this response, as estradiol and progesterone still induced a small amount of Ca^2+^ production under treatment with these inhibitors.

### Estrogen Modulates Ca^2+^-Mediated Microneme Secretion

**FIGURE 4:**
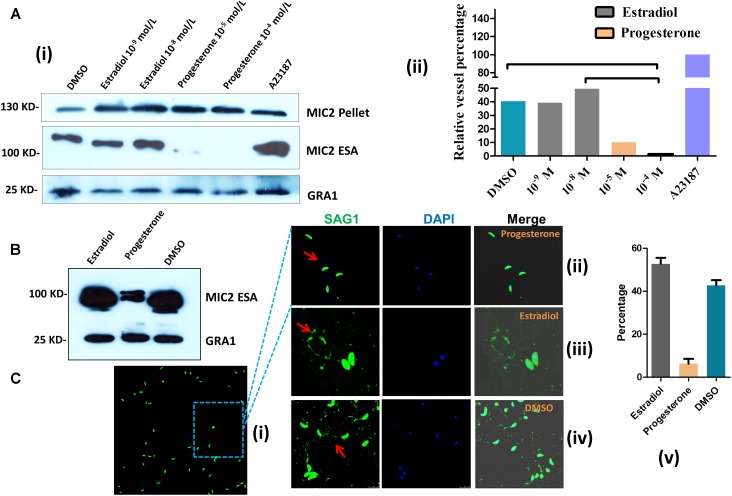
Estrogen modulates microneme secretion and gliding motility. **(A)** Western blot of parasite supernatants and lysates following treatment with estradiol or progesterone. A23187, positive control; DMSO, negative control; GRA1: control for parasite viability; ESA: excretory-secretory antigens; MIC2 and GRA1 polyclonal antibodies were prepared in our laboratory. **(i)** Western blot; **(ii)** statistical results of western blot. **(B)** Secretion of MIC2 was stimulated by estradiol or progesterone. The MIC2 monoclonal antibody was provided by Silvia N. J. Moreno, University of Georgia. **(C)** Gliding traces of *T. gondii* recorded from a representative field of view. Gliding motility is blocked by treatment with progesterone (10^-5^ mol L) compared with the estradiol (10^-8^ mol L) and DMSO groups. The SAG1 polyclonal antibody was prepared in our laboratory. **(i,ii)** progesterone pretreated *T. gondii*; **(iii)** estradiol pretreated parasites; **(iv)** DMSO pretreated parasites; **(v)** statistical results of gliding traces.

Micronemes, which are secretory organelles located at the apical end of the parasite, depend on Ca^2+^ fluxes and are essential for *T. gondii* motility (Carruthers et al., 2010). To evaluate whether estrogen is related to microneme secretion, we examined the discharge of the microneme protein MIC2. Parasites treated for 15 min with estradiol showed a slight concentration-dependent increase in MIC2 (**Figure 4A**). However, progesterone-induced MIC2 secretion (∼100 kDa, excretory-secretory antigens, ESA) was inhibited. Additionally, we found that progesterone did not inhibit MIC2 expression (∼130 kDa), as there was no observed decrease in pellets compared with those in the DMSO group. Using a monoclonal MIC2 antibody, we found that the mature form of the MIC2 protein (∼100 kDa) was significantly decreased in progesterone-pretreated parasites compared with that in the estradiol and DMSO pretreatment groups (**Figure 4B**). From the above experiments, we established that estrogen might modulate parasite invasion by modulating MIC2 protein secretion, as knocking out the MIC2 protein caused *T. gondii* to lose its invasion ability (Huynh and Carruthers, 2006).

The *T. gondii* invasion process depends on parasite motility, and parasite motility is regulated by IC Ca^2+^ fluxes (Huynh et al., 2003; Lovett and Sibley, 2003). We next examined whether estrogen treatment increased *T. gondii* motility as well. Parasite motility was analyzed by allowing parasites to glide on a substratum in the presence or absence of estrogen and then visualizing the trails produced by parasite movement via immunofluorescence (Hakansson et al., 1999). Parasites pretreated with progesterone for 30 min produced fewer and shorter trails compared with the control parasites (**Figure 4Cii**), and most *T. gondii* were in a motionless state (**Figure 4Ci**). We then counted the percentage of parasites that could form complete loop motion trails. Under our experimental conditions, estradiol incubation increased parasite motility in a manner that was somewhat comparable to DMSO incubation (**Figures 4Ciii–v**). These findings indicated that estradiol treatment increased parasite motility, which likely depended on the alteration of Ca^2+^ fluxes in the parasites. To our surprise, progesterone inhibited the gliding of *T. gondii* and reduced MIC2 protein secretion.

### Estradiol and Progesterone Treatment Induces Parasite Egress

**FIGURE 5:**
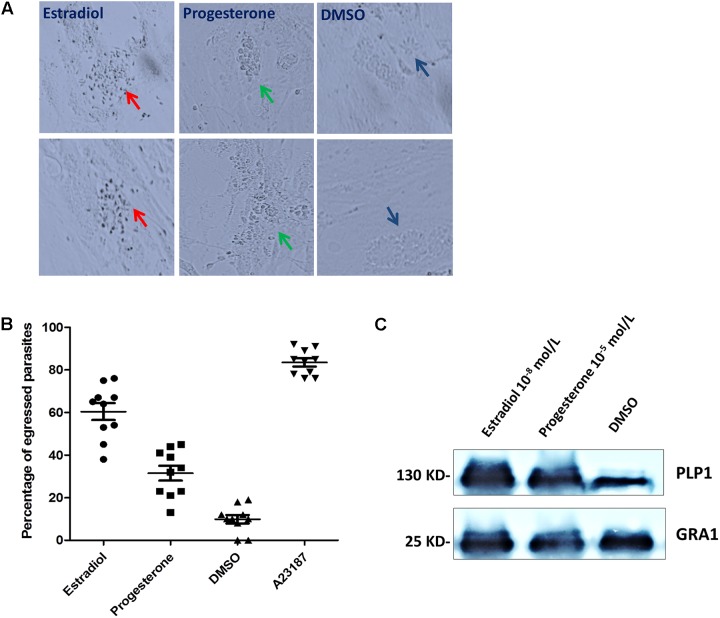
Estradiol and progesterone induce parasite egress from host cells. **(A)** HFFs were infected with *T. gondii* for approximately 36 h, followed by treatment with progesterone or estradiol. Red arrows, estradiol pretreated *T. gondii*; Green arrows, progesterone pretreated parasites; Mazarine arrows, DMSO pretreated parasites. **(B)** Statistical analysis of the percentage of egressed parasites. Error bars represent ± *SD* for 100 vacuoles counted in triplicate from three biological replicates. A23187, positive control; DMSO, negative control. **(C)** Progesterone and estradiol enhanced egress-related PLP1 protein expression compared with the DMSO group. The PLP1 polyclonal antibody was prepared in our laboratory.

We observed that estrogen treatment of extracellular *T. gondii* affected parasite motility; hence, we reasoned that estrogen might promote active egress of *T. gondii* from host cells, which is triggered by changes in the ionic environment (Moudy et al., 2001). When infected parasites were treated for 30 min with estradiol, a sharp increase in parasite egress was observed compared with that in the DMSO group (**Figure 5A**), whose egress rate was consistently observed to be 60% (**Figure 5B**). In contrast to the results of estradiol treatment, the egress rate of progesterone-pretreated parasites was low, albeit still higher than that of the control group at 33% (**Figure 5B**). However, we observed that most of the egressed parasites induced by progesterone did not migrate to neighboring cells due to low gliding motility (**Figure 5A**). In addition, progesterone inhibited the maturation of the MIC2 protein, as shown in **Figure 4**, and we can speculate that progesterone may make it more difficult for egressed *T. gondii* to invade new cells for survival. In contrast, estradiol enhanced the vitality of *T. gondii*.

PLP1 (perforin-like protein 1) was found to be necessary for rapid egress, and PLP1-deficient parasites failed to egress rapidly from the PV after calcium ionophore treatment (Roiko and Carruthers, 2013). Therefore, we detected the expression of PLP1 in *T. gondii* pretreated with progesterone and estradiol and found that the two estrogens enhanced the expression of PLP1 and promoted the egress of *T. gondii* (**Figure 5C**).

### Estrogen Does Not Affect NO and ROS Production in *T. gondii* Tachyzoites

**FIGURE 6:**
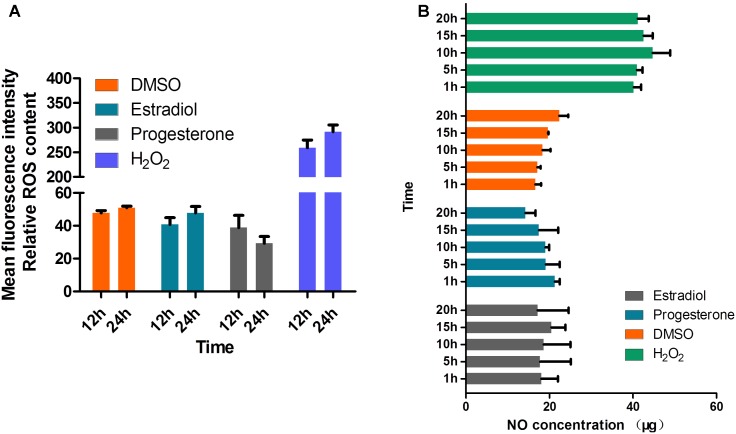
Production of NO and ROS is not regulated by estradiol and progesterone in *T. gondii.* **(A)** ROS levels in parasites were detected after continuous treatment with estradiol, progesterone, H_2_O_2_ or DMSO for 24 h. H_2_O_2_, positive control; DMSO, negative control. **(B)** Estradiol and progesterone had no effect on the NO production of parasites.

Estrogens have been reported to prevent IC peroxide accumulation, decrease ROS production (Culmsee et al., 1999), limit lipid peroxidation (Wang et al., 2006; Rattanajarasroj and Unchern, 2010), and regulate the release of nitric oxide (NO) (Skarsgard et al., 1997). One of the host mechanisms for blocking *T. gondii* replication is the production of NO. In several *in vitro* studies, NO was shown to be involved in the killing of IC *T. gondii* (Khan et al., 1997). Therefore, we suspected that estrogen may affect the invasion and survival of *T. gondii* by regulating the production of NO and ROS. However, our results showed that estrogen did not affect NO and ROS production in the parasites, even after constant observation for 24 h (**Figures 6A,B**).

## Discussion

Estrogen is associated with *T. gondii* invasion and proliferation, which has been discussed in our previous work, and Ca^2+^ signaling is crucial in parasite biology. Therefore, estrogen-induced Ca^2+^ fluxes may play an important role in the survival of *T. gondii*. In this study, we extend our understanding of the effects of estrogen on *T. gondii* by demonstrating that PKG and PI-PLC activities are important for the robust Ca^2+^ response elicited by estrogen. Furthermore, we determined the source of estrogen-induced Ca^2+^ to be distinct from different stores and used this phenomenon as the basis for explaining the relationship of estrogen with parasite gliding motility, microneme secretion, and egress.

Previous studies have indicated that Ca^2+^ is generated via modulation of the phosphoinositide-signaling pathway through binding to a surface receptor, most commonly a GPCR. However, in the present study, we found that BSA-coupled estradiol could not stimulate Ca^2+^ production. This result may indicate that GPCR-like membrane receptors did not mediate the observed Ca^2+^ release but that it occurred directly through estradiol entering the cytoplasm of *T. gondii*. Therefore, the pathway of estradiol-induced *T. gondii* Ca^2+^ fluxes remains to be further studied, and an estradiol receptor responsible for mediating IC calcium increases may reside in the cytoplasm of parasites.

Previous reports have shown that the cyclic nucleotide second messenger cyclic GMP (cGMP) activates PKG and plays a vital role in the activation of apicomplexan egress and motility (Eaton et al., 2006; Taylor et al., 2008). Inhibition of PKG interrupts cGMP signaling, leading to microneme secretion defects in both *Toxoplasma* and *Plasmodium* spp. (Collins et al., 2013). Importantly, PI(4,5)P_2_ hydrolysis by PI-PLC generates IP_3_, a second messenger that is important for Ca^2+^-dependent gametocyte activation of *Plasmodium berghei* (Raabe et al., 2011) and elicits Ca^2+^ responses in intraerythrocytic asexual stages of *P. falciparum* (Alves et al., 2011). These combined findings strongly support a mechanism in which hormone-induced Ca^2+^ release is linked to the PI-PLC and PKG pathways to activate *Toxoplasma* motility and microneme secretion. Although we did not find that a GPCR-like receptor in *T. gondii* activates Ca^2+^ flux, the observed fluxes may be related to second messenger (cGMP and IP_3_) generation.

Membrane phosphatidic acid (PA) level plays a critical role in *T. gondii* microneme secretion. PA is a key factor in the microneme secretion-signaling pathway, and blocked production of PA inhibits microneme secretion, even when the release of Ca^2+^ is induced (Bullen et al., 2016). In our study, although progesterone could induce Ca^2+^ release, similar to estradiol, it inhibited the secretion and maturation of the MIC2 protein, which was likely related to the modulation of PA. Our ELISA data showed that PA production was inhibited by progesterone (data not presented), and inhibition of PA production might be the mechanism through which progesterone reduced the secretion of MIC2. Microneme proteins are key factors in parasitic invasion, and knocking out MIC2 caused *T. gondii* to lose its invasion ability. Estradiol slightly increased the secretion of MIC2, which was one factor that enhanced the pathogenicity of *T. gondii*. However, progesterone inhibited MIC2 protein maturation and secretion, which may significantly decrease the parasitic invasion ability of *T. gondii*. Although progesterone promoted the egress of parasites from host cells, *T. gondii* may have been less pathogenic because of its reduced invasiveness. Actually our previous work showed that estradiol enhances the pathogenicity of *T. gondii* (Zhang et al., 2017), and progesterone reduces the parasite’s pathogenicity (unpublished), which is consistent with our present results.

Apicomplexans lack calmodulin-dependent kinases, which are basic components of calcium signals in mammals; instead, several calcium-dependent protein kinases (CDPKs) have been found in this group (Billker et al., 2009). For example, TgCDPK1 is a key factor in microneme exocytosis (Lourido et al., 2010), whereas TgCDPK3 is essential only for egress (Garrison et al., 2012). In the present study, progesterone inhibited the secretion of MIC2 and reduced the gliding motility of parasites. However, to our surprise, progesterone still induced the egress of *T. gondii*. We speculate that progesterone may affect the function or expression of certain proteins to trigger egress, regardless of reduced motility. Moreover, egress is assisted by the release of TgPLP1, which forms pores in the PV membrane (PVM) and the host cell plasma membrane (Garg et al., 2013). Progesterone was found to increase TgPLP1 expression in our study, which may be an important prerequisite for promoting parasite egress.

Estrogen receptors are found in mitochondria, and estrogens have substantial effects on mitochondrial biogenesis and metabolism, as well as on ROS generation (Okoh et al., 2011). In our study, both estrogens had no effect on ROS production, and no direct evidence was found linking hormone-induced calcium release and mitochondria, as shown in **Figure 2C**. Some reports (Nagamune et al., 2007) have indicated that estradiol-mediated activation of IC NO synthase occurs through stimulation of the estrogen receptor and the mitogen-activated protein (MAP) kinase pathway. However, we cannot rule out MAPK involvement based on our results, because the MAPK pathway has not been fully elucidated in *T. gondii* at present. Because of this ambiguity, we cannot elaborate further.

In summary, our research reveals estrogen-induced Ca^2+^ fluxes in *T. gondii* and further explains the relationship of estradiol and progesterone-induced Ca^2+^ with parasite invasion, gliding motility, and egress. Further work will be necessary to better understand the change in the pathogenicity of *T. gondii* during pregnancy.

## Author Contributions

XZ, JL, and QL conceived and designed the experiments. XZ performed the experiments. XZ, HZ, and YF analyzed the data. QL contributed reagents, materials, and analysis tools. XZ and QL wrote the manuscript.

## Conflict of Interest Statement

The authors declare that the research was conducted in the absence of any commercial or financial relationships that could be construed as a potential conflict of interest.
